# Opt-In and Opt-Out Consent Procedures for the Reuse of Routinely Recorded Health Data in Scientific Research and Their Consequences for Consent Rate and Consent Bias: Systematic Review

**DOI:** 10.2196/42131

**Published:** 2023-02-28

**Authors:** Yvonne de Man, Yvonne Wieland-Jorna, Bart Torensma, Koos de Wit, Anneke L Francke, Mariska G Oosterveld-Vlug, Robert A Verheij

**Affiliations:** 1 Nivel, Netherlands Institute for Health Services Research Utrecht the Netherlands; 2 Leiden University Medical Centre Leiden the Netherlands; 3 Department of Gastroenterology and Hepatology Amsterdam UMC, University of Amsterdam Amsterdam Gastroenterology Endocrinology Metabolism Amsterdam the Netherlands; 4 Department of Public and Occupational Health Location Vrije Universiteit Amsterdam Amsterdam UMC Amsterdam the Netherlands; 5 Tranzo School of Social Sciences and Behavioural Research Tilburg University Tilburg the Netherlands

**Keywords:** real-world data, secondary data use, electronic health records, routine health data, consent resentativeness, consent bias, procedure, opt-in, opt-out, consent rate, representativeness

## Abstract

**Background:**

Scientific researchers who wish to reuse health data pertaining to individuals can obtain consent through an opt-in procedure or opt-out procedure. The choice of procedure may have consequences for the consent rate and representativeness of the study sample and the quality of the research, but these consequences are not well known.

**Objective:**

This review aimed to provide insight into the consequences for the consent rate and consent bias of the study sample of opt-in procedures versus opt-out procedures for the reuse of routinely recorded health data for scientific research purposes.

**Methods:**

A systematic review was performed based on searches in PubMed, Embase, CINAHL, PsycINFO, Web of Science Core Collection, and the Cochrane Library. Two reviewers independently included studies based on predefined eligibility criteria and assessed whether the statistical methods used in the reviewed literature were appropriate for describing the differences between consenters and nonconsenters. Statistical pooling was conducted, and a description of the results was provided.

**Results:**

A total of 15 studies were included in this meta-analysis. Of the 15 studies, 13 (87%) implemented an opt-in procedure, 1 (7%) implemented an opt-out procedure, and 1 (7%) implemented both the procedures. The average weighted consent rate was 84% (60,800/72,418 among the studies that used an opt-in procedure and 96.8% (2384/2463) in the single study that used an opt-out procedure. In the single study that described both procedures, the consent rate was 21% in the opt-in group and 95.6% in the opt-out group. Opt-in procedures resulted in more consent bias compared with opt-out procedures. In studies with an opt-in procedure, consenting individuals were more likely to be males, had a higher level of education, higher income, and higher socioeconomic status.

**Conclusions:**

Consent rates are generally lower when using an opt-in procedure compared with using an opt-out procedure. Furthermore, in studies with an opt-in procedure, participants are less representative of the study population. However, both the study populations and the way in which opt-in or opt-out procedures were organized varied widely between the studies, which makes it difficult to draw general conclusions regarding the desired balance between patient control over data and learning from health data. The reuse of routinely recorded health data for scientific research purposes may be hampered by administrative burdens and the risk of bias.

## Introduction

### Background: Learning Health System

Routinely recorded health data of individuals, for example, in electronic health records, are increasingly being reused in scientific research and for quality purposes. The reuse of routine health data to generate knowledge and use this knowledge in the health care delivery process is an important aspect of what is called a “learning health system” [1,2]. The development of learning health systems is important [3,4], as it allows us to learn more about individuals’ health care use and how quality of care may be improved without increasing the administrative burden for health care providers. Such knowledge can shape policy and practice, resulting in greater quality, accuracy, accessibility, and sustainability of health care systems [2,5].

### Legislation

However, the increasing importance of reusing routine health data must be balanced against the patient’s right to data protection [6,7]. In European Union countries, the General Data Protection Regulation (GDPR) provides a general legal framework for collecting and processing personal health data, which the GDPR classifies as special category data. The GDPR generally prohibits the processing of special category data, except when there is a valid legal basis of doing so. Both a lawful basis for processing, as stated in Article 6(1) of the GDPR, and a special category condition for processing in compliance with Article 9(2) of the GDPR are necessary [8].

In general, consent is the starting point when processing special category data, and consent in the sense of the GDPR must meet a range of requirements: it must be specific, freely given, informed, and unambiguous [8]. This means that the individual has voluntarily given an express statement of consent or a clear affirmative action that leaves no room for interpretation and that consent is in the context of one or more specific purposes. This is also referred to as informed consent [9]. However, the GDPR provides an exemption from the requirement to obtain informed consent when data are used for research purposes, for instance, when research is in public interest. As the GDPR also provides the option for European Union (EU) member states to maintain or introduce further conditions, including limitations, with regard to the processing of data concerning health, the exemption from obtaining consent when performing research has been implemented differently in various EU member states [10]. Although in some EU member states it may be sufficient that the research is in public interest to be able to reuse health data for research without consent [10], it is not the same in the Netherlands.

The Dutch standards for data protection when data are used for research purposes have recently been refined in the code of conduct [11]. This code serves as an important normative framework and expresses the current consensus on the relevant European and Dutch legislation. It states that the consent (acquired by the health care provider) is, in principle, the first legal basis to release pseudonymized patient data for research. However, consent is not required in the following conditions: (1) if requesting consent from an individual is not reasonable or would impose too great a burden on the patient, for instance, in case the patient is terminally ill; (2) if the request for permission cannot reasonably be expected from the health care provider, for instance, owing to the large size of the study group; or (3) if asking for permission would lead to a low or selective response or participation rate that cannot be corrected for (ie, selection or consent bias). This can threaten the representativeness of the study sample [12-15] and ultimately render the research unreliable and, therefore, impossible. If the exemption from obtaining informed consent applies, further conditions must be met, such as the research must be in public interest and that patients have not objected to the reuse of their data for research. Researchers must also ensure that adequate technical and organizational safeguards, for example, pseudonymization, are in place when using data pertaining to patients [10].

If legislation allows it, for instance, when in the Netherlands health care researchers expect a low and selective response when asking for consent, an opt-out procedure might be a good alternative to an opt-in procedure. In an opt-out procedure, an individual is presumed to consent if they do not actively refuse the reuse of health data, whereas in an opt-in procedure, patients must actively provide informed consent. Opt-out procedures may reduce the administrative burden on health care providers. In addition, if an opt-out procedure is used and provided that patients are well informed, patients still have considerable control over the data. However, even in opt-out procedures, a selective, nonrandom group of individuals might actively object to the reuse of data, resulting in consent bias. In conclusion, both in opt-in and opt-out procedures, consent rates and consent bias can become problematic [16-19]. However, the extent to which low consent rates and consent bias occur and whether opt-in and opt-out procedures differ in these respects is unknown.

Apart from the type of consent procedure implemented, other factors can influence the health data sharing preferences of patients [20]. First, whether the consent is study specific or broad. Broad consent is a form of consent in which one consent for multiple potential future research projects in a certain scope is obtained [21]. Specific consent, rather than one-off broad consent, may increase the risk of low and biased participation rates [9]. Second, whether a legal representative is involved. The involvement of such a representative can be the case for certain groups of incompetent patients, such as those with advanced dementia and those with serious mental health problems. A legal representative might be more apprehensive about providing opt-in consent on an individual’s behalf, which can lead to low study samples. Third, whether individuals are adequately informed about the research, data protection, and data governance [22]. Research has shown that the extent to which patients understand the substance of the consent request determines how likely they are to provide consent [23,24]. This indicates that the method of providing information and obtaining consent could affect consent rates.

However, the consequences of these factors on consent rates and consent bias and whether these differ between opt-in and opt-out procedures are unknown.

### Objective and Research Questions

Therefore, our review paper aims to provide more insight into the consequences of opt-in versus opt-out procedures for the secondary use of routinely recorded health data of individuals for scientific research purposes. We were particularly interested in the consequences of opt-in versus opt-out for (1) the consent rate and (2) the representativeness of the individuals who gave consent for the study population.

The specific review questions addressed are as follows:

1. What are the consequences of opt-in versus opt-out consent procedures regarding the consent rate for the reuse of health data in scientific research?
2. What are the consequences of opt-in versus opt-out consent procedures for the reuse of health data in scientific research regarding the extent to which the sample recruited is representative of the study population?
3. To what extent, within opt-in and opt-out procedures, are the consequences for (a) the consent rate and (b) the representativeness dependent on:
  - whether the consent was study specific or broad
  - whether the individual in question or a legal representative provided consent
  - the method of informing individuals and obtaining the consent

## Methods

### Design

This paper describes the results of a systematic review. The reporting was guided by the PRISMA (Preferred Reporting Items for Systematic Reviews and Meta-Analyses) statement [25].

### Search Strategy and Study Selection

The search was performed in August 2021. The databases searched were PubMed, Embase, CINAHL, PsycINFO, Web of Science Core Collection, and the Cochrane Library. Databases were searched using a predetermined strategy, as detailed in Multimedia Appendix 1.

**Figure 1:**
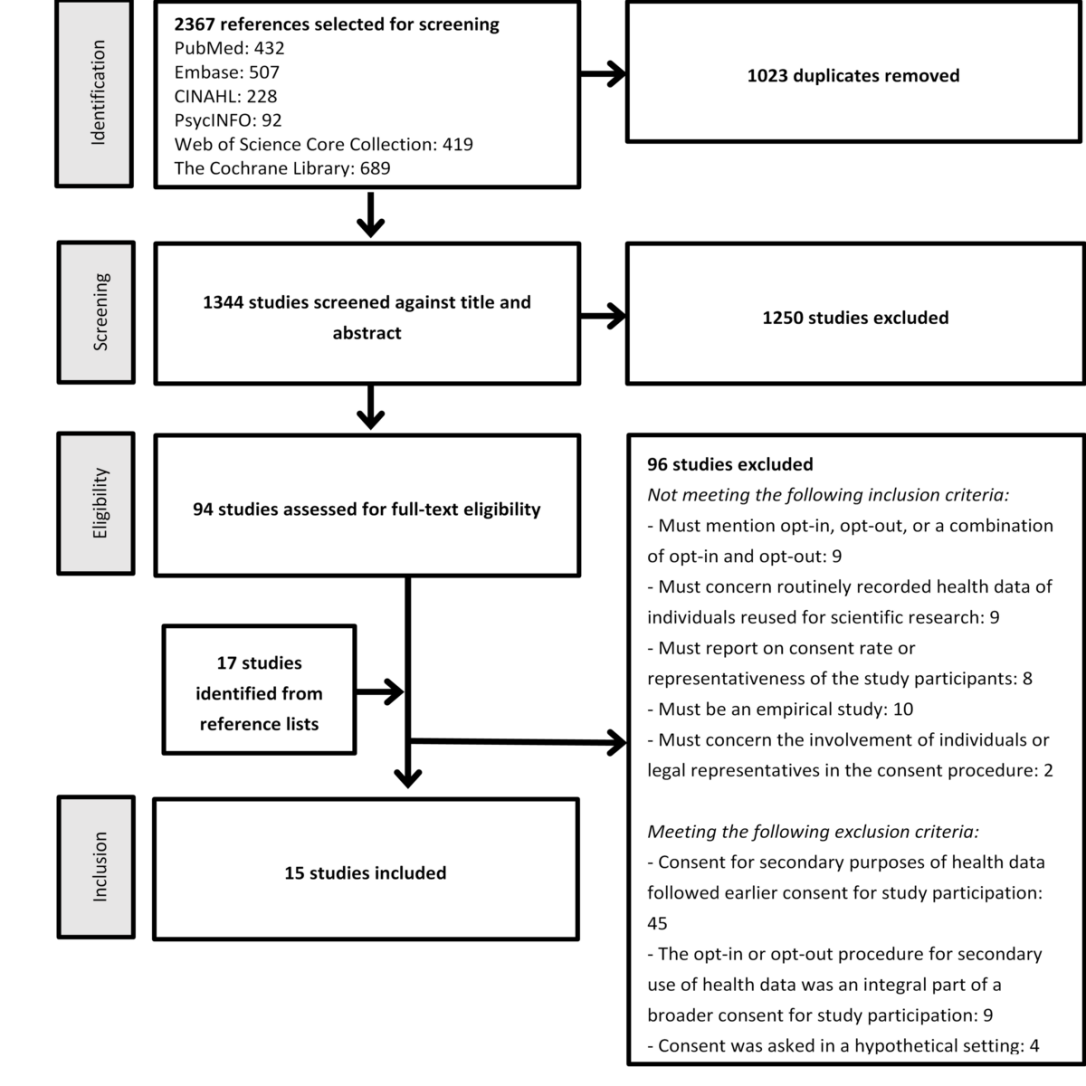
PRISMA (Preferred Reporting Items for Systematic Reviews and Meta-Analyses) flow diagram.

Two reviewers independently selected the studies that satisfied the eligibility criteria (see Inclusion Criteria). In the first step, selection was based on the title and abstract (YdM and ALF), and in the second step, the full texts were checked (YdM and YWJ or MGOV). Discrepancies in reviewer selection were resolved by discussion and consulting with another reviewer or coauthor. The selection process is described using a PRISMA flow diagram [25] (Figure 1).

### Eligibility Criteria

#### Inclusion Criteria

##### Research Participants

Included studies had to concern persons of any age who were directed in consent procedures for the reuse of individual, routinely recorded health data. These people could be patients, citizens in general, or legal representatives of the people to whom the health data pertain.

##### Research Topics

Studies must focus on the scientific research reuse of routinely recorded health data pertaining to individuals and on health data routinely recorded in relation to good clinical practice involving individuals. Studies were only included if individuals or legal representatives (any substitute decision-makers appointed under the relevant law) to whom the data pertain were involved in the consent procedure, as opposed to studies in which only the health care provider provided consent. Studies must have implemented an opt-in procedure, opt-out procedure, or a combination of opt-in and opt-out consent procedures to be included.

##### Outcome Variables

Studies were included if they provided information regarding the consent rate or information on the representativeness (in relation to the study population) of the study participants who provided consent (whether through an opt-in or opt-out procedure) for secondary use of health data.

All types of empirical studies were eligible for inclusion, including gray literature. Full texts had to be available.

#### Exclusion Criteria

Studies were excluded if the opt-in or opt-out procedures for the secondary use of health data followed earlier consent for study participation, for instance, for participation in a survey or intervention study. Studies were excluded if the opt-in or opt-out procedure for secondary use of health data was an integral part of broader consent for study participation, for instance, for participation in a survey or intervention study. Studies were also excluded if consent was obtained in a hypothetical setting (eg, the participants were asked in which situations they would be willing to provide consent for the reuse of health data containing information on their treatment).

Editorials, essays, literature reviews, and other nonempirical studies were also excluded. However, the reference lists of the relevant literature reviews were checked to identify potentially relevant empirical research. The number of excluded studies (including reasons for exclusion for studies excluded based on a review of the full text) was recorded at each selection stage in the PRISMA flow diagram.

There were no restrictions regarding publication date or language.

### Data Extraction and Synthesis

For each included study, information relevant to our review questions was extracted using a prestructured format. This information included authors and country, research questions for which routinely recorded health data were used, the setting, type of consent procedure (opt-in, opt-out, or a combination of both), whether the consent was study specific or broad, the method of obtaining consent, the person who provided consent, and the characteristics of consenters and nonconsenters.

To answer the review questions regarding consent rates, we also extracted the number of individuals approached and the number of individuals who gave consent. We pooled the extracted data regarding consent rates stratified by consent procedure (opt-in or opt-out) and the outcome of interest (broad vs specific consent, representative vs individual consent, and different methods of informing individuals). In the case of missing data regarding the numbers of individuals approached, individuals who responded, consenters, or nonconsenters, where possible, we deduced the numbers and percentages based on the numbers that were provided by the study concerned. For instance, for opt-in procedures, if the numbers of individuals who were approached and who gave consent were known, we could calculate the nonconsenters by subtracting the consenters from the individuals approached.

To answer the review questions regarding the representativeness of the study sample, we extracted and described all data describing statistical differences in characteristics between consenters and nonconsenters or differences in characteristics between consenters and the total study population. In other words, we extracted information on consent bias arising from the type of consent procedure used. We were particularly interested in the comparisons for age, sex, ethnicity, education, income, socioeconomic status (SES), and health status.

### Assessment of Statistical Quality

Two researchers (YdM and BT) independently assessed the statistical quality of the included studies, providing data for our review questions regarding the representativeness of the study population (questions 2, 2a, 2b, and 2c). The statistical quality of the descriptive data on consent rates (for questions 1, 1a, 1b, and 1c) was not assessed because descriptive statistics are straightforward and require no further statistical analyses. We assessed statistical quality by asking, “Was appropriate statistical analysis used?” The answer was scored as “yes,” “no,” or “unclear.” A “yes” was scored if (1) the study tested for differences between either consenters and nonconsenters, or consenters or nonconsenters and the study population and (2) appropriate statistical methods were used in the reviewed literature for testing the differences between consenters and nonconsenters or consenters and the study population concerning age, sex, ethnicity, education, income, SES, or health status. We judged studies to be of *good quality* if both reviewers assigned a “yes.” Any discrepancies in scoring were resolved through a consensus meeting. If a study was not assigned a “yes” by either reviewer, the extracted data regarding consent bias were excluded from the results.

## Results

### Results of the Selection Procedure

#### Overview

The searches identified 2367 study references. After removing duplicates, 1344 studies remained. After the first selection, performed by reading titles and abstracts, 94 studies were selected for full-text screening. Of these, 12% (11/94) were deemed eligible for inclusion in this review. An additional 17 potentially relevant studies were identified from the reference lists of the 11 included studies and from relevant literature reviews that were excluded in the screening phase. These 17 studies were also assessed by 2 reviewers who read the full texts. Of these, 23% (4/17) were eligible for inclusion in our review. This resulted in the inclusion of a total of 15 studies (Table 1). Figure 1 shows the PRISMA diagram.

**Table 1:** Characteristics of included studies.

Study; country	General aim or scope as described in the study	Purpose for which routine health data were obtained and analyzed	Study population	Sources and types of routine health data
Berry et al [26], 2012; Australia	To determine which approach (opt-in or opt-out) for gaining parental consent for linkage of vaccination data with hospital data provides the highest consent rate for a program of childhood vaccine safety surveillance	To examine adverse events after immunization	Children of mothers aged ≥18 years, who resided in South Australia, and who had given birth at the Women’s and Children’s Hospital between July 27, 2009, and October 25, 2009	Sources: vaccination and hospital medical records Type: vaccinations and hospital visits
Jacobsen et al [27], 1999; United States	To analyze the influence on consent rate of changes to Minnesota statutes for use of medical records for research	To achieve study aim	Sample of patients who received hospital medical care during the 3 years before January 1, 1997, aged >20 years, living in the United States	Source: hospital medical records; inpatient and outpatient Type: demographics, diagnoses, and care use
Barnes et al [28], 2005, United Kingdom	(1) To examine use, effectiveness, and tolerability of the drug montelukast for treating asthma and (2) to explore prognostic factors that could predict a favorable response to the drug	To achieve study aim	Patients (with physician-diagnosed asthma) who had been prescribed montelukast at any time between February 1998 and June 2000	Source: hospital and general practitioner medical records Type: demographics and diagnoses
Damery et al [29], 2011; United Kingdom	To establish the level of consent bias that may occur should individual patient consent be sought	To improve the understanding of the reasons for anemia	Adult patients, aged ≥40 years, who received a hematological or clinical diagnosis of iron deficiency anemia between 2001 and 2006	Source: general practitioner medical records Type: demographics and diagnoses
Elwood et al [30], 2019; New Zealand	To give a measure of consent bias resulting from consent for inclusion	To achieve study aim	All patients in New Zealand, diagnosed with a first primary invasive breast cancer or DCIS^a^ from 2000 to 2012	Source: hospital medical records Type: demographics, diagnoses, treatment, and mortality data
Evenhuis et al [31], 2004; The Netherlands	To identify unanticipated obstacles for population-based epidemiological research on visual and hearing impairment and reasons for nonparticipation	To determine the cause and degree of IDs^b^ and to assess the reliability of reported audiometric and ophthalmological data	Adults with IDs (26% of whom were diagnosed with Down syndrome) making use of ID care services	Source: hospital medical records Type: demographic and ophthalmologic and audiometric data
Jackson et al [32], 2008; United Kingdom	To assess potential consent biases arising from an opt-in procedure	To achieve study aim	All patients with a stroke or TIA^c^ admitted to the hospital or seen in outpatient clinics from October 2002 to March 2004	Source: hospital medical records (inpatient and outpatient) Type: demographics, data on process of care, and clinical variables
Knoester et al [33], 2005; the Netherlands	To evaluate whether the consent procedure induces consent bias	To conduct a retrospective chart review study of the effectiveness of lamotrigine	Patients with a first lamotrigine prescription between August 1, 1997, and December 31, 2000, aged >18 years	Source: pharmacy medical charts Type: medication
Kramer et al [34], 2017; Canada	(1) To determine success in obtaining consent from parents to allow review of their child’s personal health information for emergency research screening and (2) to examine the variables associated with successful consent	To assess future research eligibility	Parents or guardians of children aged <18 years, who presented to the study institution’s ED from July 27, 2015, to January 24, 2016, between 08:00 AM and 11:00 PM	Source: hospital ED^d^ medical records Type: demographics, diagnoses, and treatments
Marrie et al [35], 2007; United States	(1) To compare self-reported diagnoses of MS^e^ to physician-reported diagnoses, (2) to compare physician-reported diagnoses with diagnoses based on expert review of medical records, and (3) to establish markers that will identify registry participants who have a high probability of not having MS	To achieve study aim	Patients with MS included in the North American Research Committee on Multiple Sclerosis Registry	Source: hospital medical records Type: demographics and treatment
McCarthy et al [36], 1999; United States	To determine the effects of state legislation requiring the patient’s informed consent before medical record abstraction by external researchers for a study improving the potential safety of pain medication	To conduct a pharmacoepidemiological study of seizures associated with the use of pain medication	Users and nonusers of oral analgesics enrolled in the Minnesota independent practice association health plan between November 1997 and April 1998	Source: health plan administrative claims consisting of longitudinal pharmaceutical, medical, and enrollment files and hospital medical records Type: not reported
Nijhof et al [37], 2017; the Netherlands	To test potential differences between youths who refuse permission and youths who permit the use of their clinical data	To use data for benchmarking and scientific and policy research	Youth from 6 Dutch secure residential care institutions	Source: routine outcome measurements from Dutch secure residential youth care Type: demographics, diagnoses, and treatments
Noble et al [38], 2009; United Kingdom	To evaluate the effectiveness and cost of obtaining consent for a review of medical records in a study	To achieve study aim	Males aged 50-69 years with incident prostate cancer; invited between 2001 and 2008 for prostate-specific antigen testing	Source: general practitioner medical records Type: demographics and mortality data
Yawn et al [39], 1998; United States	To gather information on the number and characteristics of patients who refused authorization	To achieve study aim	All patients seen at the hospital for their first visit during January or February 1997	Source: hospital medical records (outpatient, emergency department, and inpatient) Type: demographics and diagnoses
Zell et al [40], 2000; United States	To describe the sample, the survey design, and the data collection procedures for the National Immunization Survey	To compare self-reported vaccinations with provider records	Children aged 19-35 months included in the National Immunization Survey	Sources: vaccination and hospital medical records Type: vaccinations and hospital visits

^a^DCIS: ductal carcinoma in situ.

^b^ID: intellectual disability.

^c^TIA: transient ischemic attack.

^d^ED: emergency department.

^e^MS: multiple sclerosis.

#### Main Aim or Scope

Of the studies, 67% (10/15) had a primary focus on consent rates or consent bias [26,27,29,30,32-34,36,37,39]. The 5 remaining studies were not primarily interested in consent rates or consent bias but reported consent rates as a side issue [28,31,35,38,40].

#### Study Populations

The study populations of the studies reviewed varied considerably. Although most studies focused on adults, 27% (4/15) of studies concerned health data from individuals aged <18 years [26,33,35,38].

In the following sections, the results regarding the review questions are described separately for (a) consent rates and (b) consent bias.

### Question 1: What Are the Consequences for Consent Rates of Opt-In Versus Opt-Out Consent Procedures?

#### Overview

All 15 studies reported the type of consent procedure (opt-in or opt-out) and consent rates (Table 2).

**Table 2:** Consent and nonconsent rates by consent procedure.

Consent procedure	Study	Content of consent	Approached, N	Response, n (%)	Consent, n (%)	Nonconsent, n (%)	Broad or specific consent	Method and setting for obtaining consent	Sent reminders	Consent provided by
Opt-in and opt-out	Berry et al [26], 2012	To include their medical record data in the vaccination registry (data linkage)	Opt-in: 564; opt-out: 565	Opt-in: 120 (21.3); opt-out: 25 (4.4)	Opt-in: 120 (21.3); opt-out: 540 (95.6)	Opt-in: 444 (78.7); opt-out: 25 (4.4)	Specific	Via post, signed by a pediatrician asking parents to consent or refuse via a reply form, telephone, or email	—^a^	Parents of the infants
Opt-out	Jacobsen et al [27], 1999	To review medical records for research purposes	2463	2023 (82.1)	Approximately 2384 (96.7)^b,c^ 2380^b^ (96.8)^b^	Approximately 53-79^a^ (3.2)^b^^,c^	Broad	Via post with postpaid return envelope	Reminders sent after 4, 6, and 12 weeks	Participant
Opt-in	Barnes et al [28], 2005	To review medical records for research purposes	Approximately 2500	1429 (57.2)^b^	1400 (56)	1071 (42.8)^b^	Specific	Via post	—	Participant
Opt-in	Damery et al [29], 2011	To review medical records for research purposes	592	425 (71.8)	371 (62.7)	221 (37.3)^b^	Specific	Via post with an enclosed reply slip	Reminder sent after 2 weeks	Participant
Opt-in	Elwood et al [30], 2019	To include their medical record data in the registry (data linkage)	Invasive cancer: 9244; DCIS^d^: 1642	Invasive cancer: 9244 (100); DCIS: 1642 (100)	Invasive cancer: 8282 (89.6); DCIS: 1397 (85.1)	Invasive cancer: 962 (10.4); DCIS: 245 (14.9)^e^	Broad	Verbally by a clinician during the patients’ first hospital visit	—	Participant
Opt-in	Evenhuis et al [31], 2004	To review and screen medical records	2706	2656 (98.2)	1660 (61.3)^b^	1046 (38.7)^b^	Specific	Via post through contact persons of intellectual disabilities services (usually a physician and a speech and hearing therapist or medical secretary)	Reminders sent after 1 month	Legal representative; clients who were able to communicate verbally were asked for written or verbal consent
Opt-in	Jackson et al [32], 2008	To review medical records for research purposes	1061	1061 (100)	1050 (99)	11 (1)	Broad	Inpatients: verbally after an information leaflet with a consent form was provided; outpatients: via post or during consultations after an information leaflet with a consent form was provided	—	Participant (94%) or relative in case of patients with incapacity (6%)
Opt-in	Knoester et al [33], 2005	To review medical records for research purposes	1636	1069 (65.3)	968 (59.2)	668 (40.8)	Specific	Via a recruitment letter through community pharmacists	—	Participant
Opt-in	Kramer et al [34], 2017	To review medical records for research purposes	2506	2506 (100)^b^	1852 (73.9)	654 (26.1)^b^	Specific	Verbal consent was obtained during a visit to the emergency department by a volunteer delegate	—	Parents or guardians
Opt-in	Marrie et al [35], 2007	To review medical records for research purposes	109	81 (74.3)	52 (47.7)	57 (52.3)^b^	Specific	Via post	Telephone call	Participant
Opt-in	McCarthy et al [36], 1999	To review medical records for research purposes	140	73 (52.1)	26 (18.6)	114 (81.4)^b^	Specific	Via a letter from the health plan’s medical director	Second mailing after 6 weeks and a telephone call after an additional month	Participant
Opt-in	Nijhof et al [37], 2017	To use their medical records for research purposes and benchmarking	949	887 (93.5)	628 (66.2)	316 (33.3); 5 unaccounted for by the researchers^b^	Broad	Via a questionnaire at the start of their treatment	—	Participant. However, for youth aged <16 years, the parent or legal guardian also had to consent to the data use
Opt-in	Noble et al [38], 2009	To review medical records for research purposes	193	184 (95.3)	179 (92.7)	14 (7.3)^b^	Specific	Via consent packages sent out by general practitioner practices	Reminder pack sent after 3 weeks	Participant
Opt-in	Yawn et al [39], 1998	To review medical records for research purposes	15,789	15,069^b^ (95.4)	14,493 (91.8)	1296 (8.2)^b^	Broad	Via a written consent form, as part of the hospital registration procedure with a receptionist or hospital registration clerk	—	Participant. However, if a patient had died or was aged <16 years, a legal representative was asked to sign
Opt-in	Zell et al [40], 2000	To contact providers who have administered vaccinations, who would then provide medical records for research purposes	33,344 children	33,305 (99.9) children	28,442 (85.3) children	4902 (14.7)^b^	Specific	Verbal consent was obtained at the end of a telephone interview regarding vaccination questions	—	Parents or guardians

^a^Not reported.

^b^Self-calculated values based on the numbers provided by the study.

^c^This study only reported weighted percentages. Therefore, the number of participants actively consenting and refusing was estimated and did not add up to the total number of participants who responded.

^d^DCIS: ductal carcinoma in situ.

^e^Participants who did not give consent may have declined it or may not have been offered the relevant information and consent forms; the data do not distinguish between these categories.

#### One Study Comparing an Opt-In Procedure With an Opt-Out Procedure

Berry et al [26] investigated whether consent rates differed depending on whether an opt-in or opt-out procedure was used for the same research goal. It was the only study to provide the results of both procedures. In this study, in which health data regarding children’s vaccinations and hospital visits were linked, 564 mothers were randomly assigned to the opt-in group and 565 to the opt-out group. The sociodemographic characteristics were comparable between the 2 groups. The consent rate was 21% in the opt-in group and 95.6% in the opt-out group. The differences in consent rates were statistically significant [26].

#### One Study Describing an Opt-Out Procedure

The participants in the study by Jacobsen et al [27] were actively asked for their consent for a review of hospital medical records; however, according to the Minnesota law at the time of the study, nonresponders were classified as passive consenters. Therefore, we classified this study as a study using an opt-out procedure and reported the data accordingly. In total, 2463 individuals were approached, of whom 79 (3.2%) actively refused, resulting in a consent rate of 96.8% [27].

#### Thirteen Studies Describing an Opt-In Procedure

In 13 studies, participants provided active consent for reusing health data [28-40]. In all these studies, nonresponders were classified as nonconsenters. The consent rates ranged from 18.6% to 99%. Overall, of the 72,418 individuals approached in the 13 studies, 60,831 (84%) actively gave their consent for researchers to reuse routinely recorded health data for research purposes.

To address the review questions 1a to c, we have pooled the results of the 13 studies with an opt-in procedure [28-40], together with the results of the opt-in group from the study by Berry et al [26] in which opt-in and opt-out consent rates are compared. We also grouped the results of 1 study using an opt-out procedure [27] with the results of the opt-out group from the study by Berry et al [26]. None of these studies compared an opt-in with an opt-out procedure regarding these factors potentially influencing consent; therefore, we describe the pooled results for the 2 consent procedures separately.

### Question 3a-i: What Are the Consequences for Consent Rates of Whether the Consent Procedure Is Study Specific or Broad?

Table 2 describes whether consent was study specific or broad for each study.

#### Studies With Opt-Out

In the study by Berry et al [26], consent was study specific, whereas in the study by Jacobsen et al [27], the procedure was a broad opt-out for the authorization to use medical records for research purposes in general. The 2 studies had similar consent rates (95.6% [26] vs 96.8% [27]).

#### Studies With Opt-In

Four opt-in studies obtained broad consent. The consent rates of these 4 studies ranged from 66.2% to 99%. Of the 28,685 individuals approached, 25,850 (90.1%) consented to the reuse of their health data [30,32,37,39]. This average weighted consent rate was higher than the average weighted consent rate of the 10 opt-in studies acquiring study-specific informed consent, in which a total of 44,297 individuals were approached and 35,070 (79.2%), with individual study consent rates ranging from 21.3% to 99.9%) individuals consented [26,28,29,31,33-36,38,40].

### Question 3a-ii: What Are the Consequences for Consent Rates of Whether the Individual in Question or Their Legal Representative Provided Consent?

All 15 studies reported who provided consent and consent rates (Table 2).

#### Studies With Opt-Out

In the study by Berry et al [26], parents had to opt out to refuse consent for the children’s vaccination data to be linked, whereas in the study by Jacobsen et al [27], the participants themselves had to opt out of health data reuse. However, the 2 studies had similar consent rates (95.6% [26] vs 96.8% [27]).

#### Studies With Opt-In

In the 4 opt-in studies in which a representative provided consent, a weighted average of 81.99% (32,074/39,120) approached individuals consented [26,31,34,40]. Individual consent rates ranged from 21.3% to 85.3%. For the 10 studies in which the participants provided consent, a weighted average of 85.19% (28,846/33,862) consented to the reuse of their health data, with individual study consent rates ranging from 18.6% to 99% [28-30,32,33,35-39].

### Question 3a-iii: What Are the Consequences for Consent Rates of the Method of Informing and Obtaining Consent?

All 15 studies reported on the method of informing and obtaining consent and on consent rates (Table 2).

#### Studies With Opt-Out

In the 2 studies with an opt-out procedure, the study information and an explanation of how to refuse study participation were provided by post [26,27]. The 2 studies had similar consent rates (95.6% [26] vs 96.8% [27]). Jackson et al [27] sent reminders. They did this because individuals were asked to either grant or refuse consent and to return the form in both cases. Sending reminders increased the refusal rate from 2.7% to 3.2% [27].

#### Studies With Opt-In

In the 14 studies with opt-in procedures, 4 different methods of informing and asking for consent were used, namely, verbally, mainly at the start of treatment or during a patient’s first hospital visit or stay [30,34,40]; in writing, as part of the registration or intake procedure [37,39]; by post [26,28,29,31,33,35,36,38]; or by providing an information leaflet and consent form and asking for verbal consent, or if more time was needed, the form could be returned via post [32]. For studies in which opt-in consent was requested verbally, 46,736 individuals were approached and 39,973 consented, resulting in an average weighted consent rate of 85.5%, with individual study consent rates ranging from 73.9% to 88.9%. When consent was obtained as part of the intake procedure, 90.3% (15,121/16,738) approached individuals consented, with individual study consent rates ranging from 66.2% to 91.8%. Obtaining consent via post resulted in the lowest weighted average consent rate, that is, 56.5% (8447 approached and 4776 consented), with individual study consent rates ranging from 18.6% to 92.7%. However, in the studies in which reminders were sent [29,31,35,36,38], the average weighted consent rate increased to 75.5%, with individual study consent rates ranging from 18.6% to 92.7% compared with 52.9%, with individual study consent rates ranging from 21.3% to 65.3% in the studies in which no reminders were sent [26,28,33].

### Question 2: What Are the Consequences for Consent Bias of Opt-In Versus Opt-Out Consent Procedures?

Of the studies using an opt-in or opt-out procedure, 53% (8/15) reported information on the representativeness of the study sample [26,27,29-31,33,34,37].

#### Results of the Statistical Appraisal

All 8 studies clearly described the study methodology. The study by Evenhuis et al [31] only provided descriptive statistics when comparing consenters in their study with the base population. Statistical testing for consent bias was not performed. Therefore, this study was not scored positively with regard to the statistical quality by the reviewers. The other 7 studies used various statistical analyses to assess the differences between consenters and nonconsenters, such as chi-square tests and logistic regression, and reported results including *P* values, relative risks (RRs), odds ratios, or hazard ratios. The overall consensus of the reviewers was that the statistical analyses regarding the assessment of potential consent bias were appropriate for these 7 studies.

#### Results Regarding Representativeness

The 7 studies reporting on representativeness and of good statistical quality are the study by Jacobsen et al [27] in which an opt-out procedure is described, the study by Berry et al [26] in which both an opt-in procedure and an opt-out procedure are described, and 5 studies in which an opt-in procedure is described [29,30,33,34,37]. These 7 studies reported varying consent rates, ranging from 21.3% to 97.8%, and reported comparisons between consenters and nonconsenters. The studies compared age, sex, ethnicity, education, income, SES, and health status. Al though Berry et al [26] compared opt-in and opt-out parental consent rates for childhood vaccine safety surveillance using data linkage and tested for significance, they did not test whether the 2 procedures (opt-in and opt-out) differed statistically significant with regard to consent bias. Therefore, we could only report differences between consenters and nonconsenters for the opt-in group and the opt-out group separately. In the following section, we describe the comparisons for the 6 opt-in procedures [26,29,30,33,34,37] and the 2 opt-out procedures [26,27] separately. Table 3 presents the data extracted from these outcomes.

**Table 3:** Representativeness of the study samples regarding age, ethnicity, education, income, socioeconomic status (SES), and health status or comorbidity.

Consent procedure	Study	Age	Sex	Ethnicity and migration background	Education	Income	SES	Health status and comorbidity
Opt-in and opt-out	Berry et al [26], 2012	Opt-in: *consenters are* *older*^a^; Ref^b^: 18-24 years; 25-29 years: RR^c^ 1.81 (95% CI 0.77-4.25); *P*=.17; 30-34 years: RR 3.02 (95% CI 1.38-6.61); *P*=.006; 35-39 years: RR 3.36 (95% CI 1.52-7.44); *P*=.003; ≥40 years: RR 4.16 (95% CI 1.77-9.81); *P*=.001	Opt-in: NS^d^	—^e^	Opt-in: *consenters are more highly educated*; Ref: up to year 10 (aged approximately 16 years); up to year 12: RR 1.68 (95% CI 0.64-4.36); *P*=.29; trade or certificate: RR 1.90 (95% CI 0.79-4.58); *P*=.16; university or higher: RR 3.37 (1.46-7.81); *P*=.005	Opt-in: *consenters have higher income*; Ref: <Aus $20,800 (US $13,345); Aus $20,800-$41,599 (US $13,345-$28,689): RR 0.87 (95% CI 0.40-1.85); *P*=.71; Aus $41,600-$83,199 (US $28,689-$57,374): RR 1.49 (95% CI 0.78-2.82); *P*=.23; >Aus $83,200 (US $57,374): RR 2.04 (95% CI 1.08-3.87); *P*=.03	Opt-in: NS	—
Opt-in and opt-out	Berry et al [26], 2012	Opt-out: NS	Opt-out: *fewer males participate*; RR 0.9 (95% CI 0.81-0.99); *P*=.04	—	Opt-out: NS	Opt-out: NS	Opt-out: NS	—
Opt-out	Jacobsen et al [27], 1999	*Nonconsenters are older (≥60 years);P*<.001	NS	—	—	—	—	NS
Opt-in	Damery et al [29], 2011	*Nonconsenters are older*; Ref: 40-60 years; ≥*60 years:* OR^f^ 2.84 (95% CI 2.01-4.02); *P*<.001	*Nonconsenters more likely to be female*; OR 1.62 (95% CI 1.13-2.34); *P*=.008	—	—	—	*Nonconsenters more likely to be deprived*; Ref: affluent; deprived: OR 1.61 (1.15-2.26); *P*=.005	Colorectal cancer status: NS
Opt-in	Elwood et al [30], 2019	*Consenters are younger*; ≤49 years 8.4% (231/2517) 95% CI 7.3-9.5 nonconsented; 50-69 years: 9.3% (423/4192) 95% CI 8.4-10.3 nonconsented; 70-79 years: 10.6% (109/993) 95% CI 8.5-12.7 nonconsented; 80+ years: 26.5% (199/590) 95% CI 21.6-31.4 nonconsented	—	*Varied over the strata*; adjusted for age, compared with NZ^g^ European females (9.9% nonconsented) the nonconsenting proportion was similar in Maori females (9.8%) but significantly higher in Pacific females (14.4%).: NZ European*:* 9.9% (633/5498) 95% CI 9.2-10.7 nonconsented; *Maori:* 9.8% (47/629)95% CI 6.2-13.4 nonconsented; *Pacific:* 14.4% (90/616) 95% CI 11.1-17.6 nonconsented; Asian: 12.9%; (97/708) (95% CI 9.9-15.9) nonconsented; other: 11% (39/331) 95% CI 7.4-14.7 nonconsented	—	—	—	*Nonconsenting patients had a poorer prognosis*, and 13% had metastatic disease compared with 3% of consenting patients (*P*<.001). *Nonconsenting patients more often had no primary surgery* (26.2% compared with 4% of consenting patients) and *less frequently had chemotherapy* (*P*<.001), r*adiotherapy* (*P*<.001), *hormonal therapy* (*P*<.001), or *biological therapy* (*P*<.001), in part because more nonconsenting patients declined these treatments
Opt-in	Knoester et al [33], 2005	NS	NS	—	—	NS	—	*Nonconsenters have higherCDS*^h^: Ref: CDS=0-2; *CDS>6:* hazard ratio (HR) 1.24 (95% CI 1.01-1.53); *Nonconsenters have more* *previous use of* *AEDs*^i^; Ref: 1 AED; ≥*2 AEDs:* HR 1.33 (95% CI 1.12-1.57); *Nonconsenters have more* *comedication*; Ref: absence; antidepressants*:* HR 2.01 (95% CI 1.64-2.63); antimigraine drugs: HR 1.74 (95% CI 1.16-2.61)
Opt-in	Kramer et al [34], 2007	*Child’s age:* NS	—	—	—	—	—	—
Opt-in	Nijhof et al [37], 2017	NS	NS	*Consenters more likely to be Caucasian:P*<.05; non-Western foreign: 27.1% consent versus 35.9% refuse; Western foreign: 8.1% consent versus 7.7% refuse; Caucasian: 48.4% consent versus 33.6% refuse; unknown: 16.4% consent versus 22.8% refuse	*Consenters are more highly educated:P*<.01; (special) education: 5.7% consent versus 5.4% refuse; HAVO^j^: 2.4% consent versus 1.9% refuse; MBO^k^: 7.8% consent versus 6.9% refuse; practical education: 5.9% consent versus 6.9% refuse; special higher education: 22% consent versus 10.8% refuse; VMBO^l^: 37.4% consent versus 20.5% refuse; VWO^m^: 0.8% consent versus 0.4% refuse; unknown: 18% consent versus 47.1% refuse	—	—	*Consenters have a longer treatment duration:* Consenters have 215 treatment days versus 140 for refusers; *P*<.01

^a^Text in italics is the overall conclusion as provided by the study.

^b^Ref: reference group.

^c^RR: relative risk.

^d^NS: not statistically significant.

^e^Not reported.

^f^OR: odds ratio.

^g^NZ: New Zealand.

^h^CDS: chronic disease score.

^i^AED: antiepileptic drug.

^j^HAVO: school of higher general secondary education.

^k^MBO: senior secondary vocational education.

^l^VMBO: preparatory secondary vocational education.

^m^VWO: preuniversity education.

#### Studies With Opt-Out

Jacobsen et al [27] and Berry et al [26] reported the characteristics of consenters and nonconsenters by *age*. Jacobsen et al [27] found that nonconsenters were more likely to be older than consenters. Berry et al [26] found no age-related differences between consenters and nonconsenters.

Both studies also reported the characteristics of consenters and nonconsenters by *sex*. Berry et al [26] found that fewer males gave their consent (RR 0.9, 95% CI 0.81-0.99). Jacobsen et al [27] reported no significant differences between the sexes.

Only Berry et al [26] compared consenters with nonconsenters by *educational level, SES,* and *income.* They found no significant differences related to these variables.

Jacobsen et al [27] compared consenters with nonconsenters by *health status*; they found no significant differences for this variable. None of the studies reported information regarding ethnicity [26,27].

#### Studies With Opt-In

All 6 studies with an opt-in procedure reported analyses by *age*. In the study by Berry et al [26], the consenters were older. In 2 other studies [29,30], consenters were found to be younger or nonconsenters were found to be older. Knoester et al [33] found no differences in age, and Kramer et al [34] and Nijhof et al [37] similarly reported no significant differences between consenters and nonconsenters by age.

Four of the studies with opt-in, which recruited both males and females, reported on *sex* [26,29,33,37]. In the study by Damery et al [29], females were less likely to provide consent than males. The other 3 studies reported no differences in females’ odds of consenting compared with males [26,33,37].

Elwood et al [30] and Nijhof et al [37] reported analyses of *ethnicity*. In the New Zealand clinical breast cancer registry study by Elwood et al [30], the nonconsenting proportion was similar in Maori females (9.8%) but significantly higher in Pacific females (14.4%) compared with European New Zealand females (9.9%) [30]. In the Dutch youth residential study by Nijhof et al [37], Caucasian youth were more likely to consent to health data reuse.

Berry et al [26] and Nijhof et al [37] reported differences by *educational level*. Both the studies found that consenters had a higher level of education than nonconsenters.

Berry et al [26] and Knoester et al [33] compared consenters and nonconsenters by *income*. Berry et al [26] showed that consenters were more likely to be in the highest annual household income bracket than nonconsenters (RR 2.04; CI 1.08-3.87). Knoester et al [33] found no significant difference in income.

Berry et al [26] and Damery et al [29] reported data on *SES*. Damery et al [29] reported that patients living in more deprived areas were significantly more likely not to give their consent for access to medical records than those living in more affluent areas. Berry et al [26] found no significant differences in the socioeconomic quintiles for consenters versus nonconsenters.

Of the 4 studies with opt-in that reported on *health status* [29,30,33,37], Damery et al [29] showed that colorectal cancer status was not significantly associated with consent in patients with anemia. Nijhof et al [37] showed that consenting youth in residential care institutions were more likely to have a longer treatment duration. However, in this study, consent was obtained during intake and assessed retrospectively. In the 2 remaining studies [30,33], poorer health status was reported in nonconsenters. Elwood et al [30] showed that, compared with consenters, nonconsenters had a poorer prognosis and were more likely to have a metastatic disease. In addition, nonconsenting patients less frequently received chemotherapy, radiotherapy, hormonal therapy, or biological therapy (all *P*<.001), partly because more nonconsenting patients declined these treatments. In the second study, nonconsenters were more likely to have a chronic disease score (CDS) >6 compared with consenters [33]. CDS is a measure of chronic disease status derived from population-based automated pharmacy data. A higher CDS is associated with a poorer health status [41]. Furthermore, the previous use of >2 antiepileptic drugs was more likely for nonconsenters than for consenters. Finally, the use of antidepressants and antimigraine drugs was significantly associated with nonconsent.

### Questions 3b-i-3b-iii: What Are the Consequences for Consent Bias of Whether the Consent Was Study Specific or Broad, Whether the Representative or the Individual in Question Provided Consent, and the Methodology for Obtaining Consent?

There were no associations reported between the degree of consent bias and whether study-specific consent or broad consent was obtained (review question 2a), or whether the individual to whom the data were related or the legal representative gave the consent (review question 2b). In addition, no associations were reported in the reviewed literature between the degree of consent bias and the method of informing people about the reuse of health data and of obtaining consent (review question 2c).

## Discussion

### Principal Findings

This review paper addresses the consequences of opt-in and opt-out procedures for the consent rate and the representativeness of the study sample when reusing routinely recorded health data in scientific research. We reviewed 13 studies using an opt-in procedure, 1 study using an opt-out procedure, and 1 study using both opt-in and opt-out procedures. Overall, consent rates were lower in the 14 studies with an opt-in procedure compared with the 2 studies in which an opt-out procedure was implemented. In 3 studies with opt-in, no statistically significant differences were reported between consenters and nonconsenters in terms of age, sex, or income. However, in the studies that did report significant differences, an opt-in procedure resulted in a study sample with a higher proportion of males; a higher level of education, income, and; and better health status than the study population, thereby introducing a risk of biased and invalid study results. No statistically significant differences were reported between the 2 studies with an opt-out procedure for education, income, and health status. Although significant differences in age and sex were found between consenters and nonconsenters in 1 opt-out study, no statistically significant differences were reported in the other. This seems to indicate that participants in studies with an opt-out procedure are more likely to be representative of the study population than those with studies with an opt-in procedure.

This review also aimed to examine whether differences in consent rates and representativeness for studies with opt-in versus studies with opt-out were affected by (1) the implementation of a study-specific consent procedure versus broad consent procedure, (2) different methods for informing individuals and obtaining consent, and (3) whether the individual to whom the routinely recorded health data pertained or a legal representative provided consent for the reuse of the data in scientific research.

First, our results indicate that in opt-in studies, consent rates were higher in studies that implemented a broad consent procedure than in studies that obtained study-specific consent. Approaching individuals repeatedly for consent, as would be the case when study-specific consent is required, has the disadvantage that it can potentially result in *consent fatigue*. Although more research is needed regarding this phenomenon in the context of reusing health data for scientific research, consent fatigue may lead to participants taking less time to understand the consent request [42], endangering the concept of informed consent. Obtaining consent repeatedly can also potentially lower an individual’s willingness to provide consent, leading to fewer study samples and an increased risk of consent bias. Therefore, broad consent would be a good alternative to study-specific consent, provided it would be allowed by national legislation.

Second, our review indicates that the method for informing individuals and asking for their consent affects the consent rates for opt-in procedures. Participants can be informed through different methods, such as via post or email, during their consultation with the health care provider, or as part of the registration or intake procedure. For studies using an opt-in procedure, informing individuals via post resulted in the lowest response and consent rates. Nevertheless, informing participants by post or email has a considerable potential advantage in terms of a low workload for health care professionals, as it can be done by secretariats. In addition, our results imply that consent rates can be further increased by sending reminders via post or email. However, even sending reminders does not increase the consent rate to the rate seen for opt-in procedures in which participants are asked verbally for their consent. Moreover, obtaining consent verbally in opt-in procedures still does not deliver the consent rates seen in the included studies with opt-out procedures in which individuals were recruited by post. We argue that this could be because each element of extra effort required from participants to provide consent in an opt-in or opt-out procedure could be expected to reduce consent rates and thereby increase consent bias [43]. This holds especially for opt-in procedures, as this procedure requires the participant to take action to give their consent, for example, to return a consent form by post to the researcher. Another reason may be that obtaining consent verbally gives the health care provider the possibility to give additional information easily to the patient and frame the information in a certain way, potentially steering the patient’s choice to opt in or opt out [44].

Thus far, the research literature has mainly explored the effects of specific methods for asking individuals to participate in a survey or randomized controlled trial (RCT), whereas the literature on methods to increase consent rates in opt-in or opt-out procedures to reuse routinely recorded health data is limited. Edwards et al [45], for instance, reported that frequent contact between individuals requesting consent and the research subjects increased response rates in survey research. Although the main aim of the studies in our review was not to describe the effect of specific methods for obtaining consent (eg, by post or verbally) on response rates, we argue that a high response rate is a prerequisite for achieving a high consent rate. In contrast, Caldwell et al [46] found in their systematic review that how, when, and by whom information was provided when recruiting patients for an RCT was not associated with the response rates. Instead, the content of the information was more important. Individuals’ awareness of the scope of the research and the potential health benefits increase consent rates [46]. Therefore, we deem it important that, both in opt-in and opt-out procedures, potential participants have every chance to fully understand the implications of the reuse of data pertaining to them.

Factors influencing consent rates might be further explained by the choice architecture theory, which describes how the way of presenting and describing the choice options can affect the decision maker’s choice [43]. Relevant techniques described by this theory refer to the way information is presented, such as simplifying it and framing it in a certain way, which we have mentioned previously. In addition, the arrangement of options can influence choice behavior, for instance, the setting of a default. Research has shown that, for many decisions, people accept the default [47-49]. However, whether this *default effect* also holds true when it comes to the reuse of sensitive health data for research is not known.

Third, in studies with an opt-in procedure, consent rates were slightly lower when a legal representative was requested to provide consent in the case of children’s health data or health data pertaining to patients with intellectual disabilities, rather than the individual in question. In contrast, no differences in consent rates were found for the 2 studies using an opt-out procedure. This indicates that opt-in studies involving a legal representative for children or patients with intellectual disabilities might be more prone to low sample sizes. On the basis of the 2 opt-out studies, an opt-out procedure could be a good alternative; however, this is based on 2 studies only and must thus be interpreted with caution. Further research is needed to confirm this conclusion. However, a representative will also be involved in some other groups. In groups such as people with advanced dementia, frail nursing home residents, and people with serious mental or intellectual disorders [24], individuals are often unable to make informed choices [50]. Thus far, little is known about the decision-making process of legal representatives in opt-in or opt-out procedures for the reuse of routinely recorded health data. We believe that an explanation for the lower consent rates of legal representatives is that they are more afraid or cautious regarding potential risks, such as violation of privacy, when deciding whether to give consent to someone else, especially when this person is vulnerable. Thus, specific attention must be paid to groups in which legal representatives, rather than the individuals themselves, are involved in opt-in or opt-out procedures. In addition, our findings could indicate that an opt-out procedure might be more suitable for these specific populations to prevent low study sample size and consent bias.

Overall, the results of our systematic review contribute to the ethical debate on individuals’ control of the health data that pertains to them versus the benefits to society [51,52]. Laws such as the GDPR in EU member states, which regulate the processing of sensitive data pertaining to individuals, including routine health data, aim to protect individuals’ privacy and reduce the risk of misuse of the data [10]. The question is that at what point the efforts needed to obtain a sufficient consent rate and to create a representative study sample are proportionate given (1) the privacy risks that reusing the data impose on participants and (2) the benefits that data reuse has for society. In the Introduction section, we mentioned that the conditions under which the implementation of an opt-out procedure applies are not straightforward. A better understanding of how the benefits, the aforementioned efforts, and the risks can be weighed against one another can help further define the conditions under which an opt-out or opt-in procedure is preferred.

### Strengths, Limitations, and Recommendations for Further Research

To the best of our knowledge, this is the first systematic review assessing the consequences of opt-in procedures versus opt-out procedures for consent rates and consent bias in research where health data routinely recorded by health care professionals are reused. Another strength of our review is that we used a broad search strategy in 6 literature databases and performed a statistical quality assessment of the included studies.

However, regarding our results, the following limitations should be considered. First, only 2 studies implemented an opt-out procedure to obtain authorization for the reuse of routinely recorded health data. Therefore, our results must be interpreted with caution, and further research is required to validate the results. An explanation for the low number of studies with an opt-out procedure in our review might be that opt-out procedures are not commonly used when a study reuses routinely recorded health data, as it appears to be far from straightforward to meet the legal conditions for an opt-out procedure. Another reason is that overall consent rates and consent bias might not be seen as major issues in opt-out procedures. Therefore, research exploring the consequences of opt-out procedures on the consent rate and consent bias when reusing routinely recorded health data might be limited. We recommend that future research explore these consequences further and explore how novel models of consent and informing patients affect consent rates and representativeness. Models such as electronic and dynamic consent are important strategies that may improve transparency and patients’ trust in the reuse of health data [21,53-55]. None of the studies included in this review used such a strategy.

The second limitation is that only 1 study directly compared an opt-in procedure and an opt-out procedure using an RCT design and tested for significant differences in consent rates. Extending this methodology is recommended for further research, as this could provide greater insight into whether an opt-in procedure or opt-out procedure is preferred in terms of consent rate and representativeness. Third, the pooled and weighted consent rates calculated for research question 1 were unadjusted estimates. In addition, the consent rates between the different opt-in studies varied greatly, potentially owing to the large heterogeneity between the different studies. This heterogeneity of the samples limits our ability to assess the effects of our grouping factors further. Further research on these issues is necessary to strengthen the indications. Fourth, 73% (11/15) of reviewed studies reused data recorded in a hospital setting. We suggest further empirical research to explore whether the results of our review will be similar in other settings. In addition, almost all studies involved patients diagnosed with 1 specific condition. Only 4 studies examined the general population. Patients with a specific diagnosis will probably be more engaged with a study topic related to their illness. This is likely to increase their tendency to consent to reusing health data. Whether this is the case cannot be determined from our results; therefore, we recommend further studies focusing on consent procedures in more general health settings to assess the effect of this on consent rates and consent bias.

### Conclusions

Our results indicate that consent rates are lower in studies using opt-in procedures for the secondary use of routinely recorded health data in scientific research than in studies using opt-out procedures. In addition, in studies using an opt-in procedure, consenters are more likely not to be representative of the study population compared with studies using an opt-out procedure. Specifically, consenters are more likely to be male; have a higher level of education, income, SES; and have better health status. This could imply that people with poor health or other vulnerable conditions are underrepresented in studies using opt-in procedures. In addition, our review seems to indicate that implementing study-specific consent, as opposed to broad consent, can potentially decrease consent rates in opt-in studies. In addition, the method of informing individuals and asking for their consent potentially affects consent rates. In opt-in procedures, a more direct approach and sending reminders seem to lead to higher consent rates. In studies using an opt-in procedure, consent rates were slightly lower when a legal representative was asked to provide consent in the case of children’s health data or health data pertaining to patients with intellectual disabilities, rather than the individual in question. At the same time, no differences were found in studies that used an opt-out procedure. However, both the study populations and the way in which opt-in or opt-out was organized varied widely between the studies, which makes it difficult to draw general conclusions. More research comparing opt-in and opt-out procedures and more knowledge regarding the consequences (benefits and efforts) of opt-in versus opt-out procedures are needed so that researchers can weigh up all the consequences when they want to reuse routinely recorded health data for scientific research.

## Appendix

Multimedia Appendix 1 Database search details.

## Abbreviations

CDS: chronic disease score
EU: European Union
GDPR: General Data Protection Regulation
PRISMA: Preferred Reporting Items for Systematic Reviews and Meta-Analyses
RCT: randomized controlled trial
RR: relative risk
SES: socioeconomic status
