# Supplementary material for: Opt-In and Opt-Out Consent Procedures for the Reuse of Routinely Recorded Health Data in Scientific Research and Their Consequences for Consent Rate and Consent Bias: Systematic Review
Source: J Med Internet Res. 2023 Feb 28;25:e42131. doi: 10.2196/42131 (PMC10015347; doi:10.2196/42131)
Supplement: Multimedia Appendix 1 [file jmir_v25i1e42131_app1.docx]

# APPENDIX A

### Results August 6, 2021

| **Databases** | **Result** |
| --- | --- |
| PubMed | 432 |
| Embase | 507 |
| Web of Science | 419 |
| APA PsycInfo | 92 |
| Cinahl | 228 |
| Cochrane Library | 689 |
| **Total** | **2367** |
| **After deduplication** | **1344** |

### PubMed History and Search Details August 6, 2021

| **Set** | **PubMed Query** | **Results** |
| --- | --- | --- |
| #5 | #1 AND #2 AND #3 | 432 |
| #3 | "Selection Bias"[MeSH Terms] OR "Bias"[MeSH Terms] OR "bias*"[tiab] OR "sampling error*"[tiab] OR "representative*"[tiab] OR representation[tiab] OR underrepresentation[tiab] OR "response rate*"[tiab] OR “non-respons*”[tiab] | 708,207 |
| #2 | "Informed Consent"[Mesh] OR opt-in[tiab] OR opted-in[tiab] OR opt-out[tiab] OR opted-out[tiab] OR consent*[tiab] | 111,197 |
| #1 | "Medical Records"[Mesh] OR "Routinely Collected Health Data"[Mesh] OR “medical record*”[tiab] OR “health data”[tiab] OR “health record*”[tiab] OR “patient record*”[tiab] OR “clinical registr*”[tiab] OR “clinical data”[tiab] OR “patient portal*”[tiab] OR “administrative data”[tiab] OR “real world data”[tiab] OR “routine data”[tiab] OR “routinely collected”[tiab] OR “data sharing”[tiab] OR “data link*”[tiab] OR “secondary data analys*”[tiab] OR “data reuse”[tiab] OR “secondary use”[tiab] OR “secondary analys*”[tiab] OR “data mining”[tiab] | 438,518 |

### Embase.com History and Search Details August 6, 2021

| **Set** | **Embase.com Query** | **Results** |
| --- | --- | --- |
| #5 | #4 NOT 'conference abstract'/it | 507 |
| #4 | #1 AND #2 AND #3 | 1,124 |
| #3 | 'selection bias'/exp OR 'statistical bias'/exp OR bias*:ab,ti,kw OR 'sampling error*':ab,ti,kw OR 'representative*':ab,ti,kw OR representation:ab,ti,kw OR underrepresentation:ab,ti,kw OR 'response rate*':ab,ti,kw OR 'non-respons*':ab,ti,kw | 868,593 |
| #2 | 'informed consent'/exp OR 'opt in':ab,ti,kw OR 'opted in':ab,ti,kw OR 'opt out':ab,ti,kw OR 'opted out':ab,ti,kw OR consent*:ab,ti,kw | 221,961 |
| #1 | 'health data'/exp OR 'medical record'/exp OR 'routinely collected health data'/exp OR 'medical record*':ab,ti,kw OR 'health data':ab,ti,kw OR 'health record*':ab,ti,kw OR 'patient record*':ab,ti,kw OR 'clinical registr*':ab,ti,kw OR 'clinical data':ab,ti,kw OR 'patient portal*':ab,ti,kw OR 'administrative data':ab,ti,kw OR 'real world data':ab,ti,kw OR 'routine data':ab,ti,kw OR 'routinely collected':ab,ti,kw OR 'data sharing':ab,ti,kw OR 'data link*':ab,ti,kw OR (secondary NEAR/2 (analys* OR use)):ab,ti,kw OR 'data reuse':ab,ti,kw OR 'data mining':ab,ti,kw | 826,632 |

### Cinahl (Ebsco) History and Search Details August 6, 2021

| **Set** | **Cinahl (Ebsco) Query** | **Results** |
| --- | --- | --- |
| S4 | S1 AND S2 AND S3 | 228 |
| S3 | MH ("Selection Bias+" OR "Bias (Research)+") OR TI (bias* OR “sampling error*” OR “representative*” OR representation OR underrepresentation OR “response rate*” OR “non-respons*”) OR AB (bias* OR “sampling error*” OR “representative*” OR representation OR underrepresentation OR “response rate*” OR “non-respons*”) | 181,414 |
| S2 | MH ("Consent (Research)" OR "Consent") OR TI (“opt in” OR “opted in” OR “opt out” OR “opted out” OR consent*) OR AB (“opt in” OR “opted in” OR “opt out” OR “opted out” OR consent*) | 51,677 |
| S1 | MH ("Medical Records+" OR "Electronic Health Records+" OR "Routinely Collected Health Data" OR "Electronic Data Interchange+" OR "Patient Record Systems" OR "Clinical Information Systems+" OR "Health Information Systems+") OR TI (“medical record*” OR “health data” OR “health record*” OR “patient record*” OR “clinical registr*” OR “clinical data” OR “patient portal*” OR “administrative data” OR “real world data” OR “routine data” OR “routinely collected” OR “data sharing” OR “data link*” OR (secondary N2 (analys* OR use)) OR “data reuse” OR “data mining”) OR AB (“medical record*” OR “health data” OR “health record*” OR “patient record*” OR “clinical registr*” OR “clinical data” OR “patient portal*” OR “administrative data” OR “real world data” OR “routine data” OR “routinely collected” OR “data sharing” OR “data link*” OR (secondary N2 (analys* OR use)) OR “data reuse” OR “data mining”) | 229,012 |

### APA PsycInfo(Ebsco) History and Search Details August 6, 2021

| **Set** | **APA PsycInfo (Ebsco) Query** | **Results** |
| --- | --- | --- |
| S4 | S1 AND S2 AND S3 | 92 |
| S3 | DE "Response Bias" OR TI (bias* OR “sampling error*” OR “representative*” OR representation OR underrepresentation OR “response rate*” OR “non-respons*”) OR AB (bias* OR “sampling error*” OR “representative*” OR representation OR underrepresentation OR “response rate*” OR “non-respons*”) OR KW (bias* OR “sampling error*” OR “representative*” OR representation OR underrepresentation OR “response rate*” OR “non-respons*”) | 283,848 |
| S2 | DE "Informed Consent" OR TI (“opt in” OR “opted in” OR “opt out” OR “opted out” OR consent*) OR AB (“opt in” OR “opted in” OR “opt out” OR “opted out” OR consent*) OR KW (“opt in” OR “opted in” OR “opt out” OR “opted out” OR consent*) | 25,082 |
| S1 | DE ("Medical Records" OR "Client Records" OR "Electronic Health Records") OR TI (“medical record*” OR “health data” OR “health record*” OR “patient record*” OR “clinical registr*” OR “clinical data” OR “patient portal*” OR “administrative data” OR “real world data” OR “routine data” OR “routinely collected” OR “data sharing” OR “data link*” OR (secondary N2 (analys* OR use)) OR “data reuse” OR “data mining”) OR AB (“medical record*” OR “health data” OR “health record*” OR “patient record*” OR “clinical registr*” OR “clinical data” OR “patient portal*” OR “administrative data” OR “real world data” OR “routine data” OR “routinely collected” OR “data sharing” OR “data link*” OR (secondary N2 (analys* OR use)) OR “data reuse” OR “data mining”) OR KW (“medical record*” OR “health data” OR “health record*” OR “patient record*” OR “clinical registr*” OR “clinical data” OR “patient portal*” OR “administrative data” OR “real world data” OR “routine data” OR “routinely collected” OR “data sharing” OR “data link*” OR (secondary N2 (analys* OR use)) OR “data reuse” OR “data mining”) | 49,143 |

### Web of Science Core Collection History and Search Details August 6, 2021

| **Set** | **Web of Science Core Collection Query** | **Results** |
| --- | --- | --- |
| #4 | #1 AND #2 AND #3 | 419 |
| #3 | TS = (bias* OR “sampling error*” OR “representative*” OR representation OR underrepresentation OR “response rate*” OR “non-respons*”) | 1,533,554 |
| #2 | TS = (“opt in” OR “opted in” OR “opt out” OR “opted out” OR consent*) | 93,072 |
| #1 | TS= (“medical record*” OR “health data” OR “health record*” OR “patient record*” OR “clinical registr*” OR “clinical data” OR “patient portal*” OR “administrative data” OR “real world data” OR “routine data” OR “routinely collected” OR “data sharing” OR “data link*” OR (secondary NEAR/2 (analys* OR use)) OR “data reuse” OR “data mining”) | 364,506 |

### The Cochrane Library History and Search Details August 6, 2021

| **Set** | **The Cochrane Library Query** | **Results** |
| --- | --- | --- |
| #4 | #1 AND #2 AND #3 | 689 |
| #3 | (bias* OR (sampling NEXT error*) OR “representative*” OR representation OR underrepresentation OR (response NEXT rate*) OR (non-respons*)):ti,ab,kw (Word variations have been searched) | 96,602 |
| #2 | (“opt in” OR “opted in” OR “opt out” OR “opted out” OR consent*):ti,ab,kw (Word variations have been searched) | 98,049 |
| #1 | ((medical NEXT record*) OR “health data” OR (health NEXT record*) OR (patient NEXT record*) OR (clinical NEXT registr*) OR “clinical data” OR (patient NEXT portal*) OR “administrative data” OR “real world data” OR “routine data” OR “routinely collected” OR “data sharing” OR (data NEXT link*) OR (secondary NEAR/2 (analys* OR use)) OR “data reuse” OR “data mining”):ti,ab,kw (Word variations have been searched) | 41,081 |
